# Exploring disease perception in Behçet’s syndrome: combining a quantitative and a qualitative study based on a narrative medicine approach

**DOI:** 10.1186/s13023-023-02668-8

**Published:** 2023-03-18

**Authors:** D. Marinello, I. Palla, V. Lorenzoni, G. Andreozzi, S. Pirri, S. Ticciati, S. Cannizzo, A. Del Bianco, E. Ferretti, S. Santoni, G. Turchetti, M. Mosca, R. Talarico

**Affiliations:** 1 Rheumatology Unit, Azienda Ospedaliero Universitaria Pisana, Via Roma 67, 56126 Pisa, Italy; 2 Institute of Management, Scuola Superiore Sant’Anna, Pisa, Italy; 3 Associazione S.I.M.B.A (Associazione Italiana Sindrome e Malattia di Behçet), Pontedera, Italy; 4 Rheumatology Unit, University of Pisa, Pisa, Italy

**Keywords:** Behçet’s syndrome, Disease perception, Narrative medicine, Cluster analysis, Text analysis

## Abstract

**Background:**

Behçet Syndrome (BS) has a significant psychological and social impact on patients, caregivers and families. The present study aims at exploring disease perception in BS patients, using both a co-designed survey and the narrative medicine (NM) approach.

**Methods:**

An ad-hoc questionnaire was co-designed by clinicians expert in BS, BS patients and caregivers and BS adult patients were invited to answer the online questionnaires. Cluster analysis was used to analyse data from the survey and to identify groups of patients with diverse disease perception. To further explore real-life perspectives, the stories of illness of a smaller group of adult BS patients were anonymously collected online and analysed by means of text, sentiment and qualitative analysis.

**Results:**

Two hundred and seven patients answered the survey and forty-three stories were collected. The cluster analysis highlighted that accepting or not the disease has a strong impact on the daily life, on how BS patients perceive themselves and in terms of hope for the future. The stories revealed that patients often address common issues, such as the long and complex journey faced from the disease onset until the BS diagnosis, which was strongly connected to the concept of time and perceived as an exhausting period of their lives.

**Conclusion:**

To our knowledge, this is the first study that addressed disease perception also applying the NM principles in BS. The current perception that BS patients have of their disease should encourage the BS scientific and patient community in joining forces in order to improve the journey of BS patients.

**Supplementary Information:**

The online version contains supplementary material available at 10.1186/s13023-023-02668-8.

## Background

Behçet syndrome (BS) is a rare, chronic and multisystemic disorder affecting mucosa, skin, joints, eyes, nervous and gastrointestinal system. The multi-organ involvement and the wide range of clinical spectrum make often the management of BS challenging; moreover, the relapsing course of the disease can determine exacerbations and remission of symptoms over time [1].

Various demographic factors, such as age at disease onset, duration of disease or gender, are considered predictive of poor outcomes in the short and long-term. In fact, younger male patients have a more severe disease, leading to increased morbidity and mortality [2].

BS has a significant psychological and social impact on the patients, on their caregivers and families. In routine clinical practice, BS patients frequently describe to have experienced several emotions such as fear, anxiety, stress, depression and anger because of the difficulty to adapt their lives to the disease, as well as uncertainty about their future [3–6]. Furthermore, patients sometimes highlight the difficulty to socialise due to their symptoms and in several cases, they refer that the disease negatively impacts on their familiar relationships [7, 8].

Studies exploring the experience and the patients' perception in BS are few; literature data are mainly focused on Quality of Life (QoL) in terms of depression, anxiety and sleep quality, while less data are available on the qualitative evaluation of QoL. The qualitative assessment of the patient’s perception of the disease, however, is very important to have a holistic approach to the disease and support patient centered therapeutic and management decisions. From a qualitative study performed in New Zealand exploring the experience and the challenges of a small group of patients living with BS, some important challenges emerged such as the difficulty to obtain a correct and timely diagnosis, loneliness and isolation due to the rarity and the difficulty to interact with the healthcare system [9].

Therefore, the present study is aimed at exploring disease perception among a large community of Italian BS patients, by means of a co-designed survey and applying the narrative medicine (NM) approach [10–12] collecting stories of BS patients.

## Objectives

The main objectives of the study were: (i) to evaluate disease perception in a large community of BS adult patient; (ii) to identify eventual clusters of BS patients with different perception of disease; (iii) to explore areas affecting disease perception that are not captured with conventional assessment, through patients’ stories collected using the NM approach.

## Methods

### Study design and population

A cross-sectional study was conducted to investigate disease perception among adult Italian BS patients. In detail, two different approaches were used. On a large community of Italian BS patients, disease perception was assessed by means of an ad-hoc questionnaire developed in co-design with patients and caregivers, clinicians and other experts; the general aim was to investigate the dimensions of quality of life and disease perception in BS.

A smaller group of BS patients provided insights into disease perception using the NM approach in a separate form. Participation to the questionnaire was voluntary and anonymous and they were asked for their consent to analyse their answers for research purpose (a specific approval was asked in the introduction text of the survey). For anonymous surveys only notification of the Ethical Committee of the University of Pisa is needed, which deemed formal IRB approval unnecessary.

### Measures

An ad-hoc questionnaire was co-designed in Italian by clinicians expert in the management of BS, health economists, patients’ representative and caregivers in collaboration with the Italian Association for Behçet Disease (SIMBA OdV) [13]. The questionnaire was implemented online using EUSurvey [14] and promoted among Italian BS patients trough different dissemination channels with the support of SIMBA OdV that contributed to the dissemination of the survey (i.e., website, Social media, etc.). Participation to the questionnaire was voluntary and data were collected from July 2019 to October 2019-point Likert-scale questions and were explored asking patients about the impact of the disease on different aspects of their life (i.e., work, family, social relations, etc.).

In order to further explore real-life perspectives of BS patients, the NM approach was adopted and the stories of illness of BS patients were anonymously collected online from September 2019 to December 2019. In details, a semi-structured questionnaire was developed to capture the demographic profile of the respondents (Table 1), while a wider section was dedicated to guide patients in telling their stories (e.g. “How did you feel when you were diagnosed with BS?”, “Did you experience issues in informing your employer about your disease?”, “Do you feel at the centre of your care? Which are your needs and expectations for the future?”), for which 3600 characters were available.

**Table 1:** Main characteristics of the study population of the disease perception and QoL survey

	N	%
*Gender*		
Female	139	67.15
Male	68	32.85
*Age*		
18–20 years	8	3.86
21–30 years	30	14.49
31–40 years	66	31.88
41–50 years	71	34.3
51–60 years	26	12.56
61–70 years	6	2.9
*Age at first symptoms*		
0–10 years	37	17.87
11–20 years	55	26.57
21–30 years	57	27.54
31–40 years	44	21.26
41–50 years	13	6.28
51–60 years	1	0.48
*Time since diagnosis*		
< 1 years	29	14.01
1–5 years	62	29.95
6–10 years	41	19.81
11–15 years	28	13.53
16–20 years	21	10.14
> = 21	26	12.56
*Marital status*		
Single	61	29.47
Married	96	46.38
Cohabitant	27	13.04
Divorced	22	10.63
Widow	1	0.48
*Education*		
None	1	0.48
High school diploma	109	52.66
Secondary school diploma	33	15.94
Degree	42	20.29
Postgraduate degree	22	10.63
*Working condition*		
Housewife	11	5.31
Unemployed	24	11.59
Unable to work	17	8.21
Retired	9	4.35
Student	15	7.25
Employed	131	63.29
*Full-time/Part-time worker*		
Part-time	37	28.24
Full time	94	71.76
*Need to change working life*		
No	71	34.30
Yes	136	65.70

### Statistical analysis

Data collected with the survey were first analysed using standard descriptive statistics, considering mean and standard deviation to describe quantitative variables and frequency for categorical variables. A cluster analysis was performed to identify possible subgroups of the overall study population based on the variables related to disease perception collected through the survey and using an approach specifically dedicated to the analysis of mixed continuous and categorical data. In particular, the method adopted is based on the application of the partitive k-medoid method, which consists of iteratively grouping the most similar units. Given the nature of the variables, the method was applied to the matrix of dissimilarity between the calculated variables using Gower's distance. The optimal number of clusters was determined on the basis of the Silhouette index.

A descriptive analysis of variables used in the cluster analysis and the main socio-demographics characteristics of patients grouped into the different cluster identified was performed in order to explore differences between clusters not only in terms of variables contributing to cluster identification, the Fisher exact test and the Chi-square test were used to assess differences among clusters.

Before performing the analysis of patients’ stories with dedicated software, pre-processing and cleaning of the texts (i.e., removing punctuation, converting all text to lowercase, removing unnecessary terms such as articles) as well as tokenization of the words by breaking up the texts into discrete words were performed. In order to explore the main words used and the concepts expressed in the stories, a word frequency analysis was also completed, and results were presented through a word cloud image. In addition, a sentiment analysis was also performed using the *get_nrc_sentiment* function implemented in the syuzhet R package [15], emotions expressed within stories collected were identified and scored according to Saif Mohammad’s National Research Council (NRC) Emotion lexicon [16]. Basically, the NRC associates the retrieved text words with eight emotions: anger, fear, anticipation, trust, surprise, sadness, joy, and disgust. Total score for each emotions detected was reported. All analyses were performed using R version 3.6.2 and *p* value < 0.05 was considered statistically significant. Considering the narrative nature of the stories, a further deep qualitative analysis was performed by experts in narrative medicine with the aim of identifying the emergent topics (both needs and experiences) and to explore the most personal and specific characteristics related to living with BS.

## Results

### Analysis of data from the survey

A total of 207 patients participated in the survey and the main characteristics of participants are detailed in Table 1. Patients answering the survey were mainly female (67.15%) and the majority of them were aged between 31 and 50 years (66.18%). About 63% of patients were employed and about 66% also declared they had the need to change their working life due to BS.

With respect to the disease, time since diagnosis was largely variable while almost all patients experienced the first symptoms before 40 years of age.

Table 2 details results related to the questions specifically related to disease perception and QoL.

**Table 2:** Characteristics of the study population related to disease perception

	N	%
*Is the therapy you are taking keeping your illness under control?*		
1-Not at all	13	6.28
2	37	17.87
3	76	36.71
4	52	25.12
5-Completely	29	14.01
*Do you feel guilty towards people close to you because of your health condition?*		
Never	21	10.14
Rarely	28	13.53
Sometimes	76	36.71
Often	59	28.5
Always	23	11.11
*Have you experienced apprehension, concern or fear for your health?*		
Never	10	4.83
Rarely	30	14.49
Sometimes	78	37.68
Often	66	31.88
Always	23	11.11
*Does the rarity of your illness make you feel lonely?*		
Never	36	17.39
Rarely	39	18.84
Sometimes	61	29.47
Often	56	27.05
Always	15	7.25
*Do you feel understood by the people around you?*		
Never	14	6.76
Rarely	46	22.22
Sometimes	66	31.88
Often	54	26.09
Always	27	13.04
*Does your illness have economic consequences on your life?*		
1-Not at all	23	11.11
2	33	15.94
3	45	21.74
4	50	24.15
5-A lot	56	27.05
*Do you have a caregiver?*		
No	118	57
Yes	89	43
*Do you have the perception that your illness is unpredictable?*		
Never	1	0.48
Rarely	10	4.83
Sometimes	69	33.33
Often	78	37.68
Always	40	23.67
*Is it easy to live with your illness?*		
Never	38	18.36
Rarely	65	31.4
Sometimes	75	36.23
Often	26	12.56
Always	3	1.45
*Do you think you can do something to improve your symptoms?*		
Never	102	49.28
Sometimes	70	33.82
Often	28	13.53
Always	7	3.38
*Has your illness affected the way others see you?*		
1-Not at all	29	14.01
2	25	12.08
3	71	34.3
4	47	22.71
5-Absolutely yes	35	16.91
*Do you feel ashamed of your illness?*		
Never	91	43.96
Rarely	38	18.36
Sometimes	46	22.22
Often	24	11.59
Always	8	3.86
*Do you think the disease has changed you?*		
No	11	5.31
Yes	163	78.74
Not completely	33	15.94
*Can you talk openly about your illness?*		
Never	7	3.38
Rarely	22	10.63
Sometimes	56	27.05
Often	49	23.67
Always	73	35.27
*Do you think your illness will get worse over time?*		
1-Not at all	4	1.93
2	15	7.25
3	71	34.3
4	58	28.02
5-Absolutely yes	59	28.5
*Do you think you have accepted the fact that you have Behçet's disease?*		
No	17	8.21
Yes	120	57.97
Not completely	70	33.82
*Has your illness affected your perception of yourself?*		
1-Not at all	14	6.76
2	19	9.18
3	71	34.3
4	46	22.22
5-Absolutely yes	57	27.54
*Do you feel worried about your health?*		
Never	5	2.42
Rarely	26	12.56
Sometimes	83	40.1
Often	71	34.3
Always	22	10.63
*Do you think you get sick more easily than others?*		
Never	15	7.25
Rarely	29	14.01
Sometimes	50	24.15
Often	78	37.68
Always	35	16.91
*Has your illness had many effects on your life?*		
1-Not at all	1	0.48
2	10	4.83
3	46	22.22
4	63	30.43
5-Absolutely yes	87	42.03
*Are your family members worried about your health?*		
Never	12	5.8
Rarely	25	12.08
Sometimes	57	27.54
Often	75	36.23
Always	38	18.36
*Has your illness had an impact on your family?*		
1-Not at all	12	5.8
2	29	14.01
3	49	23.67
4	55	26.57
5-Absolutely yes	62	29.95
*Do you know other people suffering from Behçet's disease?*		
No	71	34.3
Yes	136	65.7
*Are you aware of the existence of a Behçet's disease patient association?*		
No	10	4.83
Yes	197	95.17
*Are you in contact with or have you used the services of the association?*		
No	84	40.58
Yes	123	59.42

Globally, answers to questions related to disease perception and QoL showed some degree of variability, while it emerged that most patients reported some concerns with respect to their health status and the impact of BS on their life. In details, 76% (n = 158) of patients declared to feel guilty towards people close to them because of their health condition “Sometimes” to “Always”, with the same frequency, 81% (n = 167) of patients experiencing apprehension, concern or fear for their health.

The fact that BS can be very unpredictable is perceived almost unanimously among the study population and 90% (n = 187) felt the unpredictability of BS “Sometimes” to “Always”; moreover, 49% (n = 102) felt they can’t do anything to improve their symptoms.

BS was perceived to substantially affect how patients perceive themselves (n = 174, 84%) and that the disease has changed them (n = 163, 79%); the disease was reported to have an impact on the life of the patients, determining moderate to significant economic consequences in about 73% (n = 151) responders and also determining an impact on their family (n = 166, 80%).

Results from the cluster analysis, performed to identify groups of patients reporting diverse feelings with respect to disease perception, revealed the presence of three different groups with different attitudes towards disease perception but also characterized by some heterogeneity with respect to socio-demographics characteristics.

Details of the three groups identified are reported in Table 3 and a graphical representation of the cluster identified on a bi-dimensional plane is reported as Additional file 1: Fig. S1.

**Table 3:** Results from the cluster analysis

	Number of subjects (%)			*P* value
	CL1 (N = 75)	CL2 (N = 70)	CL3 (N = 62)	
Age				
18–20 years	2 (2.7%)	2 (2.9%)	4 (6.5%)	0.048
21–30 years	13 (17.3%)	9 (12.9%)	8 (12.9%)	
31–40 years	34 (45.3%)	16 (22.9%)	16 (25.8%)	
41–50 years	15 (20%)	30 (42.9%)	26 (41.9%)	
51–60 years	10 (13.3%)	10 (14.3%)	6 (9.7%)	
61–70 years	1 (1.3%)	3 (4.3%)	2 (3.2%)	
*Gender*				
Female	55 (73.3%)	35 (50%)	49 (79%)	0.007
Male	20 (26.7%)	35 (50%)	13 (21%)	
*Age at first symptoms*				
0–10 years	16 (21.3%)	6 (8.6%)	15 (24.2%)	< 0.001
11–20 years	24 (32%)	16 (22.9%)	15 (24.2%)	
21–30 years	24 (32%)	14 (20%)	19 (30.6%)	
31–40 years	8 (10.7%)	26 (37.1%)	10 (16.1%)	
41–50 years	3 (4%)	8 (11.4%)	2 (3.2%)	
51–60 years	0 (0%)	0 (0%)	1 (1.6%)	
*Years since diagnosis*				
< 1 years	5 (6.7%)	16 (22.9%)	8 (12.9%)	0.023
1–5 years	19 (25.3%)	25 (35.7%)	18 (29%)	
6–10 years	25 (33.3%)	7 (10%)	9 (14.5%)	
11–15 years	9 (12%)	9 (12.9%)	10 (16.1%)	
16–20 years	10 (13.3%)	5 (7.1%)	6 (9.7%)	
21–25 years	2 (2.7%)	2 (2.9%)	6 (9.7%)	
> 25 years	5 (6.7%)	6 (8.6%)	5 (8.1%)	
*Marital status*				
Single	21 (28%)	17 (24.3%)	23 (37.1%)	0.101
Married	43 (57.3%)	32 (45.7%)	21 (33.9%)	
Cohabitant	6 (8%)	12 (17.1%)	9 (14.5%)	
Divorced	5 (6.7%)	8 (11.4%)	9 (14.5%)	
Widow	0 (0%)	1 (1.4%)	0 (0%)	
*Education*				
None	0 (0%)	1 (1.4%)	0 (0%)	0.015
Secondary school diploma	8 (10.7%)	13 (18.6%)	12 (19.4%)	
High school diploma	32 (42.7%)	41 (58.6%)	36 (58.1%)	
Degree	25 (33.3%)	7 (10%)	10 (16.1%)	
Post-graduate degree	10 (13.3%)	8 (11.4%)	4 (6.5%)	
*Working condition*				
Housewife	4 (5.3%)	3 (4.3%)	4 (6.5%)	0.245
Unemployed	7 (9.3%)	10 (14.3%)	7 (11.3%)	
Unable to work	3 (4%)	2 (2.9%)	12 (19.4%)	
Retired	55 (73.3%)	47 (67.1%)	29 (46.8%)	
Student	3 (4%)	3 (4.3%)	3 (4.8%)	
Employed	3 (4%)	5 (7.1%)	7 (11.3%)	
*Quality of life*				
1-Extremely bad	0 (0%)	1 (1.4%)	11 (17.7%)	0.001
2	13 (17.3%)	12 (17.1%)	19 (30.6%)	
3	39 (52%)	38 (54.3%)	26 (41.9%)	
4	18 (24%)	18 (25.7%)	5 (8.1%)	
5-Extremely good	5 (6.7%)	1 (1.4%)	1 (1.6%)	
*Is the therapy you are taking keeping your illness under control?*				
1-Not at all	3 (4%)	4 (5.7%)	6 (9.7%)	< 0.001
2	8 (10.7%)	17 (24.3%)	12 (19.4%)	
3	18 (24%)	25 (35.7%)	33 (53.2%)	
4	32 (42.7%)	13 (18.6%)	7 (11.3%)	
5-Completely	14 (18.7%)	11 (15.7%)	4 (6.5%)	
*Do you feel guilty towards people close to you because of your health condition?*				
Never	10 (13.3%)	7 (10%)	4 (6.5%)	0.004
Rarely	12 (16%)	11 (15.7%)	5 (8.1%)	
Sometimes	24 (32%)	37 (52.9%)	15 (24.2%)	
Often	21 (28%)	12 (17.1%)	26 (41.9%)	
Always	8 (10.7%)	3 (4.3%)	12 (19.4%)	
*Have you experienced apprehension, concern or fear for your health?*				
Never	3 (4%)	6 (8.6%)	1 (1.6%)	0.017
Rarely	12 (16%)	12 (17.1%)	6 (9.7%)	
Sometimes	42 (56%)	26 (37.1%)	10 (16.1%)	
Often	12 (16%)	21 (30%)	33 (53.2%)	
Always	6 (8%)	5 (7.1%)	12 (19.4%)	
*Does the rarity of your illness make you feel lonely?*				
Never	19 (25.3%)	13 (18.6%)	4 (6.5%)	< 0.001
Rarely	24 (32%)	8 (11.4%)	7 (11.3%)	
Sometimes	21 (28%)	28 (40%)	12 (19.4%)	
Often	9 (12%)	19 (27.1%)	28 (45.2%)	
Always	2 (2.7%)	2 (2.9%)	11 (17.7%)	
*Do you feel understood by the people around you?*				
Never	2 (2.7%)	5 (7.1%)	7 (11.3%)	0.425
Rarely	17 (22.7%)	14 (20%)	15 (24.2%)	
Sometimes	23 (30.7%)	21 (30%)	22 (35.5%)	
Often	21 (28%)	19 (27.1%)	14 (22.6%)	
Always	12 (16%)	11 (15.7%)	4 (6.5%)	
*Does your illness have economic consequences on your life?*				
1-Not at all	9 (12%)	12 (17.1%)	2 (3.2%)	< 0.001
2	16 (21.3%)	16 (22.9%)	1 (1.6%)	
3	26 (34.7%)	12 (17.1%)	7 (11.3%)	
4	11 (14.7%)	17 (24.3%)	22 (35.5%)	
5-A lot	13 (17.3%)	13 (18.6%)	30 (48.4%)	
*Do you have a caregiver?*				
No	34 (45.3%)	57 (81.4%)	27 (43.5%)	< 0.001
Yes	31 (41.3%)	13 (18.6%)	35 (56.5%)	
*Do you have the perception that your illness is unpredictable?*				
Never	0 (0%)	1 (1.4%)	0 (0%)	< 0.001
Rarely	8 (10.7%)	1 (1.4%)	1 (1.6%)	
Sometimes	22 (29.3%)	34 (48.6%)	13 (21%)	
Often	26 (34.7%)	29 (41.4%)	23 (37.1%)	
Always	19 (25.3%)	5 (7.1%)	25 (40.3%)	
*Is it easy to live with your illness?*				
Never	7 (9.3%)	6 (8.6%)	25 (40.3%)	< 0.001
Rarely	20 (26.7%)	23 (32.9%)	22 (35.5%)	
Sometimes	35 (46.7%)	28 (40%)	12 (19.4%)	
Often	11 (14.7%)	12 (17.1%)	3 (4.8%)	
Always	2 (2.7%)	1 (1.4%)	0 (0%)	
*Do you think you can do something to improve your symptoms?*				
Never	29 (38.7%)	39 (55.7%)	34 (54.8%)	0.002
Rarely	37 (49.3%)	13 (18.6%)	20 (32.3%)	
Often	8 (10.7%)	15 (21.4%)	5 (8.1%)	
Always	1 (1.3%)	3 (4.3%)	3 (4.8%)	
*Has your illness affected the way others see you?*				
1-Not at all	13 (17.3%)	14 (20%)	2 (3.2%)	< 0.001
2	14 (18.7%)	9 (12.9%)	2 (3.2%)	
3	21 (28%)	31 (44.3%)	19 (30.6%)	
4	20 (26.7%)	10 (14.3%)	17 (27.4%)	
5-Absolutely yes	7 (9.3%)	6 (8.6%)	22 (35.5%)	
*Do you feel ashamed of your illness?*				
Never	40 (53.3%)	38 (54.3%)	13 (21%)	< 0.001
Rarely	11 (14.7%)	15 (21.4%)	12 (19.4%)	
Sometimes	15 (20%)	14 (20%)	17 (27.4%)	
Often	6 (8%)	3 (4.3%)	15 (24.2%)	
Always	3 (4%)	0 (0%)	5 (8.1%)	
*Do you think the disease has changed you?*				
No	4 (5.3%)	6 (8.6%)	1 (1.6%)	0.136
Yes	55 (73.3%)	53 (75.7%)	55 (88.7%)	
Not completely	16 (21.3%)	11 (15.7%)	6 (9.7%)	
*Can you talk openly about your illness?*				
Never	1 (1.3%)	1 (1.4%)	5 (8.1%)	0.012
Rarely	8 (10.7%)	5 (7.1%)	9 (14.5%)	
Sometimes	18 (24%)	16 (22.9%)	22 (35.5%)	
Often	13 (17.3%)	23 (32.9%)	13 (21%)	
Always	35 (46.7%)	25 (35.7%)	13 (21%)	
*Do you think your illness will get worse over time?*				
1-Not at all	0 (0%)	4 (5.7%)	0 (0%)	< 0.001
2	7 (9.3%)	5 (7.1%)	3 (4.8%)	
3	38 (50.7%)	19 (27.1%)	14 (22.6%)	
4	18 (24%)	24 (34.3%)	16 (25.8%)	
5-Absolutely yes	12 (16%)	18 (25.7%)	29 (46.8%)	
*Do you think you have accepted the fact that you have BS?*				
No	4 (5.3%)	3 (4.3%)	10 (16.1%)	< 0.001
Yes	58 (77.3%)	42 (60%)	20 (32.3%)	
Not completely	13 (17.3%)	25 (35.7%)	32 (51.6%)	
*Has your illness affected your perception of yourself?*				
1-Not at all	5 (6.7%)	6 (8.6%)	3 (4.8%)	< 0.001
2	12 (16%)	5 (7.1%)	2 (3.2%)	
3	21 (28%)	38 (54.3%)	12 (19.4%)	
4	21 (28%)	13 (18.6%)	12 (19.4%)	
5-Absolutely yes	16 (21.3%)	8 (11.4%)	33 (53.2%)	
*Do you feel worried about your health?*				
Never	7 (9.3%)	6 (8.6%)	2 (3.2%)	< 0.001
Rarely	10 (13.3%)	16 (22.9%)	3 (4.8%)	
Sometimes	20 (26.7%)	20 (28.6%)	10 (16.1%)	
Often	29 (38.7%)	24 (34.3%)	25 (40.3%)	
Always	9 (12%)	4 (5.7%)	22 (35.5%)	
*Do you think you get sick more easily than others?*				
Never	7 (9.3%)	6 (8.6%)	2 (3.2%)	< 0.001
Rarely	10 (13.3%)	16 (22.9%)	3 (4.8%)	
Sometimes	20 (26.7%)	20 (28.6%)	10 (16.1%)	
Often	29 (38.7%)	24 (34.3%)	25 (40.3%)	
Always	9 (12%)	4 (5.7%)	22 (35.5%)	
*Has your illness had many effects on your life?*				
1-Not at all	1 (1.3%)	0 (0%)	0 (0%)	< 0.001
2	2 (2.7%)	8 (11.4%)	0 (0%)	
3	21 (28%)	21 (30%)	4 (6.5%)	
4	29 (38.7%)	26 (37.1%)	8 (12.9%)	
5-Absolutely yes	22 (29.3%)	15 (21.4%)	50 (80.6%)	
*Are your family members worried about your health?*				
Never	2 (2.7%)	3 (4.3%)	7 (11.3%)	0.005
Rarely	7 (9.3%)	11 (15.7%)	7 (11.3%)	
Sometimes	31 (41.3%)	19 (27.1%)	7 (11.3%)	
Often	24 (32%)	28 (40%)	23 (37.1%)	
Always	11 (14.7%)	9 (12.9%)	18 (29%)	
*Has your illness had an impact on your family?*				
1-Not at all	5 (6.7%)	5 (7.1%)	2 (3.2%)	< 0.001
2	13 (17.3%)	13 (18.6%)	3 (4.8%)	
3	13 (17.3%)	28 (40%)	8 (12.9%)	
4	33 (44%)	12 (17.1%)	10 (16.1%)	
5-Absolutely yes	11 (14.7%)	12 (17.1%)	39 (62.9%)	
*Do you know other people suffering from BS?*				
No	13 (17.3%)	44 (62.9%)	14 (22.6%)	< 0.001
Yes	62 (82.7%)	26 (37.1%)	48 (77.4%)	
*Are you aware of the existence of a BS patient association?*				
No	1 (1.3%)	7 (10%)	2 (3.2%)	0.053
Yes	74 (98.7%)	63 (90%)	60 (96.8%)	
*Are you in contact with or have you used the services of the association?*				
No	16 (21.3%)	49 (70%)	19 (30.6%)	< 0.001
Yes	59 (78.7%)	21 (30%)	43 (69.4%)	

Cluster 1 grouped mainly young (< = 40 years) women and 80% of them had the first symptoms before 31 years; about 50% had a degree or higher level of education; the majority was convinced that therapy is able to control disease; a variable percentage felt guilty towards people close to them because of their health condition; the majority rarely or never felt lonely because of rarity of their disease; about 40% had a caregiver; the majority perceive the unpredictability of their disease often/always; more than 50% never felt ashamed of their illness; about 60% felt that their illness had an impact on their family; about 80% knew other people suffering from BS and were in contact with of the association (or used their service).

Cluster 2 comprised mainly men and women older than 40 years; more than 80% had first symptoms between 11 and 50 years; more than 50% had diagnosis in the last 5 years; the majority was neutral or not really convinced that therapy is able to control the disease. More than 60% of them felt “sometimes” or “often” concerned or fear about their health; the minority had a caregiver; more than 50% never felt that they were able to do something to improve their symptoms. In addition, more than 50% of them never felt ashamed of their illness; the majority thought their illness will get worse over time; more than 50% had family members frequently worried about their health; about 60% didn’t know other people suffering from BS.

Patients grouped in Cluster 3 were aged mainly between 21 and 50 years and being mainly women; more than 80% had first symptoms before 31 years and reported their QoL as bad or fair; 50% is neutral with respect to the assumption that therapy is able to control the disease; more than 50% often or always felt guilty towards people close to them because of their health condition, experienced concern or fear for their health, felt lonely because of their rare disease and experienced economic consequences because of the disease. More than 50% of them had a caregiver, while the majority often/always perceive the unpredictability of their disease and they had difficulty living with their illness; more than 50% never felt able to do something to improve their symptoms; the majority perceived that the illness affected the way others see them; more than 50% felt ashamed of their illness at least sometimes. Moreover, only about 40% of them was able to openly talk about their disease and the majority tough their illness will get worse over time. The large majority had not, or not completely, accepted the fact that they have BS and declared illness affected the perception of themselves; about 80% often or always felt worried about their health and thought they get sick more easily than others. In addition, almost all taught their illness frequently had many effects on your life; more than 50% had family members frequently worried about their health; about 80% felt their illness had an impact on their family; less than 80% knew other people suffering from BS and about 70% were in contact with the association (or used their service).

### Analysis of BS Patients’ stories

**Fig. 1:**
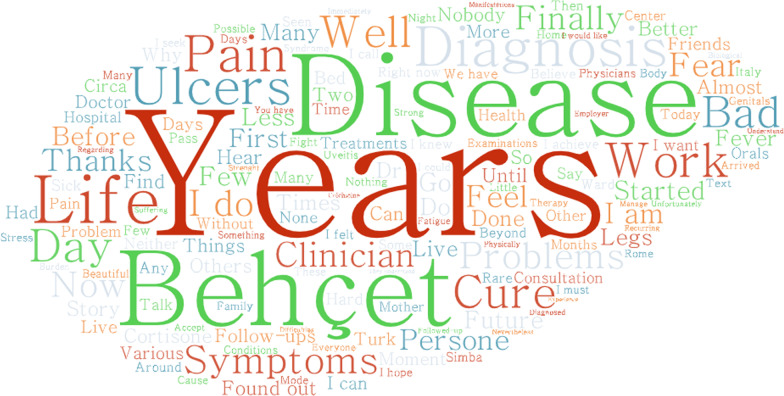
Word cloud of the most frequent terms reported in the patients’ stories. The present word cloud represents a collection of the words depicted in different sizes. The bigger and bolder the word appears, the more often it’s mentioned within the text that patients written for narrative medicine approach to disease perception

A total of 43 stories were collected from patients and their demographic data are summarised in Table 4. The most frequent words expressed in the stories, after removing articles, conjunctions and punctuations, are represented in a word cloud (Fig. 1). The most frequent words used in the stories were *years* (found n.75 times), *disease* (found n.73 times) and *Behçet* (found n.38 times), while a series of other words such as *diagnosis*, *symptoms* and *ulcers* were repeated in the stories with a similar frequency (found n.30, 29, 29 times). In addition, the words *problems*, *life* and *work* also emerged as frequent words (found n.27, 26, 26 times).

**Table 4:** Demographic data of the BS patients that contributed with their stories

Number of stories collected	43
M/F	17/26
Mean age	41

**Fig. 2:**
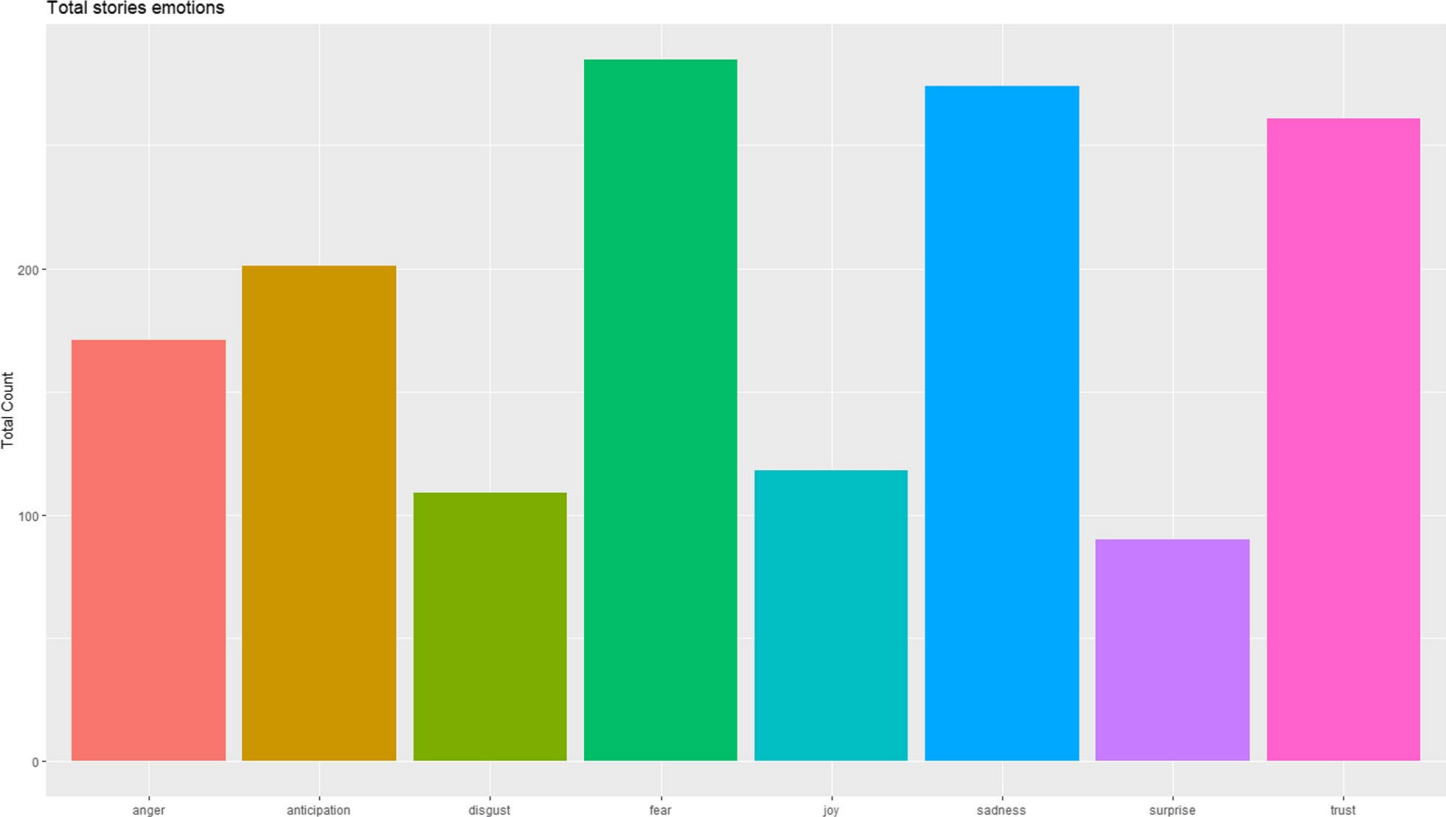
Sentiment analysis from the patients’ stories. Each column indicates a specific sentiment. This specific sentiment analysis models focus on feelings and emotions (anger, anticipation, disgust, fear, joy, sadness, surprise, trust)

The sentiment analysis showed fear and anger as the most prevalent emotions that were expressed in the stories probably with reference to the long and difficult journey lived by BS patients before getting the diagnosis as well as concerns on the different symptoms experienced. However, a sense of trust also emerged, possibly linked to the hope of having more experts centres for BS and of having a future cure for BS available for all patients (Fig. 2).

The stories provided a very deep and emotional glance into the journey of BS patients. As a matter of fact, using a qualitative approach to analyse text allow identification of feelings and perceptions related to three main phases that were related to the pre-diagnosis phase, the time of diagnosis and after the diagnosis.

*“I didn’t understand, I didn’t know”.* In the pre-diagnosis phase, patients expressed frustration and concern due to the many exams, consultations and hospitals they had to experience before getting the diagnosis. In addition, patients experienced a deep feeling of mortification when being addressed as “hypochondriac” or “depressed” and felt not understood or listened by the healthcare professionals that were treating them. The stories tell, in fact, of the many years (and money) spent by patients while travelling across the country in different hospitals searching for the right clinician that could formulate a specific diagnosis.

“*When I had my diagnosis, I felt reassured, because I knew who I was fighting*”. When receiving the diagnosis of BS, patients tell about their mixed feelings that range from confusion and rage to relief and satisfaction. Having a precise diagnosis was perceived as the beginning of a new journey, something unknown due to the uncertainty, the complexity and the rarity of BS and, parallelly, as something that brought “certainty” in the life of BS patients, “knowing who to fight” is perceived as a liberation for the dark feelings lived during the pre-diagnosis phase.

*“After all, life can be beautiful also with BS”.* After the diagnosis, patients have clearly expressed that in many cases, the new journey have brought them into a new dimension, in which personal life, relationships and working life had to realign to the new scenario. Being a BS patient brought a new awareness of themselves and their priorities, pushing them to reconsider what really matters in life and to have a new, deep sensitivity toward the outside world. In terms of personal relationships, patients report that informing their friends, families and other people close to them caused opposite reactions: on one side, people that either denied the disease or that “disappeared” and on the other side, people that understood their feelings, that provided help and continuous support, “slowing down” when patients weren’t able to face life at same speed.

Globally, an important enhancer was represented by the role played by the BS patients’ association, that was perceived as a source of information, of help and as “safe place” in which patients could share their emotions and feeling and most of all “didn’t feel alone anymore”.

## Discussion

The present study offers an overview of disease perception among adult BS patients, combining two different approaches. On one side, a first assessment was performed by means of a co-designed survey aimed at exploring both disease perception and quality of life among BS patients; on the other side, the NM approach was adopted to allow patients to freely express their feeling about the disease, thus also disclosing aspects potentially not covered with the survey.

Results from the survey revealed that, despite some degree of variability among the study population, patients generally reported some concerns with respect to the impact of BS on their life and families, also in view of the unpredictable nature of the disease. BS is also perceived to significantly affect patients’ perception of themselves and of the world around them, especially in terms of working life and personal relationships.

The cluster analysis performed in our study allowed the identification of three different groups of subjects that perceive the disease differently. The three groups were characterized by diverse feelings about their disease perception but also characterized by different socio-demographics profiles. The first group of BS patients is convinced that their treatment can control the disease and were in contact with other people affected by BS. The second group is not really convinced that the therapy is able to control BS, while about two thirds of them didn’t know anyone else affected by BS. On the other hand, a third group have not accepted the disease even if they are in contact with other BS patients. Therefore, we can assume that knowing other BS patients and being in contact with a patients’ organisation can help. However, accepting or not the disease has a strong impact not only on the daily life, but also in terms of how they perceive themselves and in terms of hope for the future.

The NM approach adopted in this study allowed to further explore individual perceptions and needs of BS patients. Despite telling their individual story, patients often addressed common issues, such as the long and complex journey faced from the disease onset until the BS diagnosis is formulated, which was strongly connected to the concept of time and perceived as an exhausting period of their lives. Data from the literature described how delays in BS is a well-known issue [17–21] and this can be aligned to the fact that many stories described in great detail the different milestones of the period lived before the diagnosis, including specificities on the hospitals visited and on the clinicians consulted.

A strong focus on emotions and feelings permitted to enter the complexity of living with BS. The combination of very different emotions perceived at the time of diagnosis highlights how important it is to ensure an early diagnosis for BS patients and to provide an appropriate flow of information on the disease when communicating the diagnosis, also taking into account the important role played by the patients’ organizations.

Although the findings are not directly comparable (due to the different methodological approaches adopted), the results of our study are partially in line with previous studies also from different countries on the impact that BS has on the lives of the patients [5–7].

To our knowledge few studies tried to get insight into patients’ perceptions using the NM approach and a structured qualitative analysis, some recent experience emerged for diseases other than BS [22, 23] none combine a quantitative and qualitative approach to deepen into disease perception among BS patients.

Some limitations of our study need to be acknowledged. First, the selected nature of the patients cannot exclude the presence of selection bias, thus limiting the generalizability of results; second, the approach used to collect answers from the survey and patients’ stories does not allow to link the answers to the questionnaire with the surveys, also preventing to know if there are patients who participated in both evaluations.

## Conclusions

To our knowledge, this is the first study on BS that addressed disease perception with a combined approach involving questionnaires co-designed with patients and narrative medicine that allows to take into account the perspectives and the experiences of BS patients. Listening to the voice of patients is really important and several methodological approaches can be adopted to do that; in fact, the main novelty of our study is represented by the combination of different approaches, such as narrative medicine, supporting the fact that the usual evidence-based medicine techniques can be integrated with different methodologies, in order to improve the understanding of the perspective of the patient. As a matter of fact, this combined approach can provide invaluable information not only for the BS community, but also for the real-life clinical practice, since having a better understanding of how the BS patient perceive the disease, also in terms of disease activity, and the impact of BS in his/her life, can definitely support the usual approaches to the disease and improve the management of BS patients.

## Supplementary Information

**Additional file 1.** Graphical representation of subjects on a bidimensional plane according to cluster membership

## Data Availability

The data collected have been reported in the results. For further information, please contact author for specific data requests.

## References

[1] Yazici Y (2020). Management of Behçet syndrome. Curr Opin Rheumatol.

[2] Hatemi G, Seyahi E, Fresko I, Talarico R, Hamuryudan V (2020). One year in review 2020: Behçet's syndrome. Clin Exp Rheumatol.

[3] Talarico R, Palagini L, Elefante E, Ferro F, Tani C, Gemignani A, Bombardieri S, Mosca M (2018). Behçet's syndrome and psychiatric involvement: is it a primary or secondary feature of the disease?. Clin Exp Rheumatol.

[4] Talarico R, Palagini L, d'Ascanio A, Elefante E, Ferrari C, Stagnaro C, Tani C, Gemignani A, Mauri M, Bombardieri S, Mosca M (2015). Epidemiology and management of neuropsychiatric disorders in Behçet's syndrome. CNS Drugs.

[5] Senusi AA, Ola D, Mather J, Mather J, Fortune F (2017). Behçet's syndrome and health-related quality of life: influence of symptoms, lifestyle and employment status. Clin Exp Rheumatol.

[6] Yankouskaya A, Boughey A, McCagh J, Neal A, de Bezenac C, Davies SJ (2019). Illness perception mediates the relationship between the severity of symptoms and perceived health status in patients with Behçet disease. J Clin Rheumatol.

[7] Ozguler Y, Merkel PA, Gurcan M, Bocage C, Eriksen W, Kutlubay Z, Hatemi G, Cronholm PF; OMERACT Behçet's Syndrome Working Group. Patients' experiences with Behçet's syndrome: structured interviews among patients with different types of organ involvement. Clin Exp Rheumatol. 2019;37(6 Suppl 121):28–34. (**Epub 2019 Apr 12. PMID: 31025933**). PMC9885438 31025933

[8] Sweeting F, Arden-Close E (2020). The impact of Behçet's disease on intimate relationships in women: a qualitative study. Chronic Illn.

[9] Tai V, Lindsay K, Sims JL, McQueen FM (2017). Qualitative study: the experience and impact of living with Behcet's syndrome. N Z Med J.

[10] Charon R (2006). Narrative medicine: honoring the stories of illness.

[11] Greenhalgh T, Hurwitz B (1999). Why study narrative?. BMJ.

[12] Hurwitz B (2000). Narrative and the practice of medicine. Lancet.

[13] https://www.behcet.it/.

[14] https://ec.europa.eu/eusurvey/.

[15] Jockers M. Package ‘syuzhet’ Type Package Title Extracts Sentiment and Sentiment-Derived Plot Arcs from Text. 2017.

[16] Mohammad SM, “NRC Emotion Lexicon.” https://saifmohammad.com/WebPages/NRC-Emotion-Lexicon.htm (Last access: July 2021).

[17] Yazici H, Seyahi E, Hatemi G, Yazici Y. Behçet syndrome: a contemporary view. Nat Rev Rheumatol. 2018;14(2):107–119. 10.1038/nrrheum.2017.208. Epub 2018 Jan 3. Erratum in: Nat Rev Rheumatol. 2018 Jan 24;14 (2):119. PMID: 29296024. 10.1038/nrrheum.2017.208 29296024

[18] Gorial FI, Jabbar MA (2020). Impact of disease activity on health related quality of life in patients with Behçet's disease: a cross-sectional study. Ann Med Surg (Lond).

[19] Can Sandikci S, Colak S, Omma A, Enecik ME (2019). An evaluation of depression, anxiety and fatigue in patients with Behçet's disease. Int J Rheum Dis.

[20] Talarico R, Marinello D, Manzo A, Cannizzo S, Palla I, Ticciati S, Gaglioti A, Trieste L, Pisa L, Badalamenti L, Randisi G, Del Bianco A, Lorenzoni V, Turchetti G, Mosca M (2021). Being a caregiver of a Behçet's syndrome patient: challenges and perspectives during a complex journey. Orphanet J Rare Dis.

[21] Marinello D, Di Cianni F, Del Bianco A, Mattioli I, Sota J, Cantarini L, Emmi G, Leccese P, Lopalco G, Mosca M, Padula A, Piga M, Salvarani C, Taruscio D, Talarico R (2021). Empowering patients in the therapeutic decision-making process: a glance into Behçet's syndrome. Front Med (Lausanne)..

[22] Palandri F, Benevolo G, Iurlo A (2018). Life for patients with myelofibrosis: the physical, emotional and financial impact, collected using narrative medicine—results from the Italian ‘Back to Life’ project. Qual Life Res.

[23] Walter MJM, van’t Spijker A, Pasma A (2017). Focus group interviews reveal reasons for differences in the perception of disease activity in rheumatoid arthritis. Qual Life Res.

