# Supplementary material for: Exploring disease perception in Behçet’s syndrome: combining a quantitative and a qualitative study based on a narrative medicine approach
Source: Orphanet J Rare Dis. 2023 Mar 18;18:58. doi: 10.1186/s13023-023-02668-8 (PMC10024433; doi:10.1186/s13023-023-02668-8)

**Supplementary Figure 1.** Graphical representation of subjects on a bidimensional plane according to cluster membership. Each colour corresponds to the different clusters observed in the study


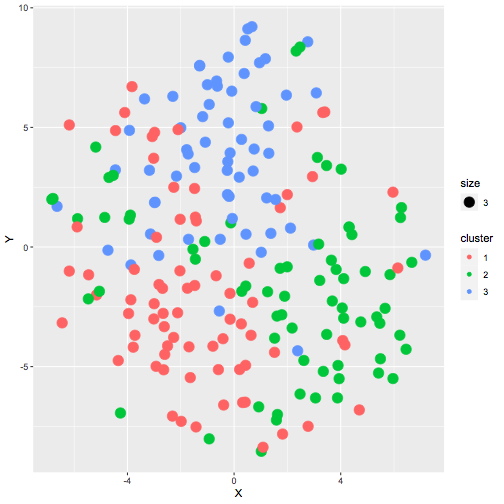

Supplement: Supplementary file 1 — Additional file 1. Graphical representation of subjects on a bidimensional plane according to cluster membership [file 13023_2023_2668_MOESM1_ESM.docx]
